# Discovery, Development, Inventions and Patent Review of Fexinidazole: The First All-Oral Therapy for Human African Trypanosomiasis

**DOI:** 10.3390/ph15020128

**Published:** 2022-01-21

**Authors:** Mohd Imran, Shah Alam Khan, Mohammed Kanan Alshammari, Ashwaq Muiedh Alqahtani, Turkiah Abdullah Alanazi, Mehnaz Kamal, Talha Jawaid, Mohammed M. Ghoneim, Sultan Alshehri, Faiyaz Shakeel

**Affiliations:** 1 Department of Pharmaceutical Chemistry, Faculty of Pharmacy, Northern Border University, Rafha 91911, Saudi Arabia; 2 College of Pharmacy, National University of Science and Technology, Muscat 130, Oman; shahalam@nu.edu.om; 3 Department of Pharmaceutical Care, Rafha Central Hospital, North Zone, Rafha 91911, Saudi Arabia; ii_kanan101@outlook.com; 4 Orange Pharmacies, Riyadh 13248, Saudi Arabia; alqahtaniashwag@gmail.com; 5 Department of Pharmaceutical Care, King Fahd Specialist Hospital, Tabuk 47717, Saudi Arabia; turkiah212@gmail.com; 6 Department of Pharmaceutical Chemistry, College of Pharmacy, Prince Sattam Bin Abdulaziz University, Al-Kharj 11942, Saudi Arabia; mailtomehnaz@gmail.com; 7 Department of Pharmacology, College of Medicine, Al Imam Mohammad Ibn Saud Islamic University (IMSIU), Riyadh 13317, Saudi Arabia; talhajawaid71@gmail.com; 8 Department of Pharmacy Practice, College of Pharmacy, AlMaarefa University, Ad Diriyah 13713, Saudi Arabia; mghoneim@mcst.edu.sa; 9 Department of Pharmaceutics, College of Pharmacy, King Saud University, Riyadh 11451, Saudi Arabia; salshehri1@ksu.edu.sa

**Keywords:** neglected disease, trypanosomiasis, fexinidazole, invention, patent review

## Abstract

Human African trypanosomiasis (HAT or ‘sleeping sickness’) is a neglected tropical disease. If untreated, it is always fatal and leads to death. A few treatments are available for HAT, but most of them require a skilled professional, which increases the financial burden on the patient. Recently, fexinidazole (FEX) has been approved by the European Medicine Agency (EMA) and the United States Food and Drug Administration (USFDA) as the first all-oral therapy for the treatment of stage-1 (hemolymphatic) as well as stage-2 (meningoencephalitic) of HAT. Before the FEX approval, there were separate treatments for stage-1 and stage-2 of HAT. This study reviews the discovery, development timeline, inventions, and patent literature of FEX. It was first approved by EMA and USFDA in 2018 and 2021, respectively. FEX was also added to the World Health Organization’s list of essential drugs in 2019. The patent literature search revealed many types of patents/patent applications (compound, salt, process, method of treatment, drug combinations, and compositions) related to FEX, which have been summarized in this article. The authors foresee a great scope to develop more inventions based on FEX (novel salts, polymorphs, drug conjugates, cyclodextrin complex, etc.) for the treatment of many protozoal diseases (Leishmaniasis and Chagas disease), inflammatory diseases, and other microbial infections. New combinations of FEX with other treatments of HAT may also provide fruitful results. This review might be useful to the scientists working on the HAT and other neglected diseases to develop novel inventions and innovations of therapeutic relevance.

## 1. Introduction

Neglected tropical diseases (NTDs) including human African trypanosomiasis (HAT or ‘sleeping sickness’), and Chagas disease (American trypanosomiasis) are a diverse set of illnesses that primarily afflict people of tropical and sub-tropical countries, which are linked to extreme poverty [1]. HAT is caused by the protozoan *Trypanosoma brucei* (*T.b.*), whereas Chagas disease is caused by *Trypanosoma cruzi* [1]. HAT is the major NTD in Africa and its presence has been recorded in about 37 countries, especially in sub-Saharan African countries [2]. However, there has been a decrease in the number of global cases of HAT per year from 1998 (about 300,000 cases) to 2009 (<10,000 cases), 2014 (<4000 cases), 2016 (about 2100 cases), and the year 2020 is thought to have <1000 cases per year. The World Health Organization also planned a total control on HAT globally by 2030 [2]. Two pathogenic sub-species of T.b. (*T.b. gambiense* and *T.b. rhodesiense*) are known [1,2]. Another subspecies (*T.b. brucei*) is considered non-pathogenic to humans and is used for research purposes because it preserves all the other features of the two pathogenic species [3].

HAT is a vector-borne disease, wherein the parasites (*T.b. rhodesiense* and *T.b. gambiense*) enter the human body through the bite of tsetse flies [4,5]. The HAT caused by *T.b. gambiense* (gHAT) (Western and Central African form) is a chronic form of the disease and is responsible for about 85–90% of cases of HAT. The signs and symptoms of gHAT appear after months or years. The HAT caused by *T.b. rhodesiense* (rHAT) (Eastern and Southern African form) is an acute form of the disease and is responsible for about 10–15% of cases [4,5,6]. The signs and symptoms of rHAT appear after 2–3 weeks. After the bite of the tsetse fly, the parasite enters the body and triggers two stages of the disease. In stage 1 (haemolymphatic stage), the parasite multiplies and spreads all over the bloodstream, systemic organs, and lymphatic system. The common non-specific symptoms of stage 1 are similar to those of the common cold, fever, headache, and joint pain [4,5]. In stage 2 (encephalitic stage) which starts after one year or later, the parasite enters the central nervous system (CNS). Stage 2 of HAT leads to chronic encephalopathy with brain damage, giving rise to neuropsychiatric disorders, poor concentration, disruption of the sleep–wake cycle, and prolonged states of drowsiness. Therefore, it is also called sleeping sickness. In the early phase of stage 2, the WBC count is slightly high in the cerebrospinal fluid (6–20 cells per µL of CSF) which subsequently increases sharply in the late phase of stage 2. The CNS infection ultimately leads to coma and the patient’s death [4,5]. The gHAT and rHAT can be diagnosed by direct visualization of the trypanosomes in blood or organ samples under a microscope, serological tests, card agglutination test, and the RDP rapid diagnostic test as SD bioline [4,5,6,7,8]. If untreated, HAT is always fatal and leads to death. The current treatment for HAT is provided in Table 1.

It is evident from Table 1 that few treatments are available for HAT, wherein nifurtimox and pentamidine are USFDA approved drugs. Most of these treatments are injectable and are effective either for stage-1 or stage-2 of HAT [9,10,11,12,13,14]. None of the available treatments are effective in treating stage-1 as well as stage-2 of HAT. Most of the treatments require a skilled person for IV administration, which increases the financial burden on the patient. In 2018, 2019, and 2021, FEX was approved by the EMA, the Democratic Republic of Congo, and USFDA for the treatment of both first-stage (hemolymphatic) and second-stage (meningoencephalitic) of HAT due to gHAT in patients 6 years of age and older and weighing at least 20 kg [15,16,17] (Table 2).

Some reviews on FEX have been published [14,17,18,19]. However, none of them is directed to the inventions and patent literature of FEX. To make new inventions and innovations related to any drug, it is crucial to understand the prior inventions related to the drug. Therefore, the authors intended to write the titled review on FEX that will be helpful to the scientists of the pharmaceutical industry/academia to generate new ideas and develop inventions related to FEX.

## 2. Fexinidazole (FEX)

**Figure 1:**
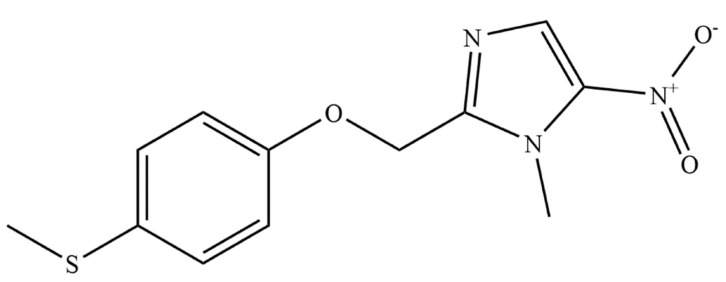
Chemical structure of fexinidazole.

FEX (Figure 1), a 2-substituted-5-nitroimidazole antiprotozoal agent, chemically is 1-methyl-2-{[4-(methylthio)phenoxy]methyl}-5-nitro-1H-imidazole (molecular formula: C_12_H_13_N_3_O_3_S: molecular weight: 279.3; CAS registry number: 59729-37-2; Other identifiers: HOE-239) [14,17,18,19]. It is a yellow crystalline, non-hygroscopic powder, which is practically insoluble in water, sparingly soluble in acetone and acetonitrile, very slightly soluble in ethanol, and slightly soluble in methanol. It is an achiral molecule and does not have optically active isomers [14,17,18,19]. It is marketed as a base, and only one polymorphic form-I is identified to date. FEX exhibits solubility and permeability characteristics consistent with BCS class II drugs [20].

**Scheme 1:**
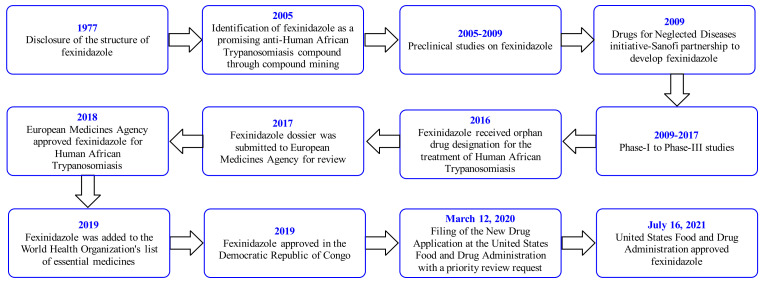
Development timeline for fexinidazole.

The structure of FEX was disclosed by Hoechst AG (Germany) in 1977 [21]. However, the company abandoned this compound due to its strategic planning. The Drugs for Neglected Diseases initiative (DNDi) was established in 2003. In 2005, the DNDi scientists and the scientists of Sanofi screened about 700 nitroheterocyclic compounds against HAT. This led to the identification of FEX (HOE239) as a promising candidate for further development [18,19]. The positive pre-clinical studies [22] on FEX led to an agreement between Sanofi and DNDi, where DNDi was responsible for the preclinical/clinical/pharmaceutical development and Sanofi was accountable for the industrialization/production/registration/distribution of the drug. FEX is the first new chemical entity developed by DND*i*. Its Phase I study was started in 2009, whereas the Phase II/III trial was started in 2012 at the Democratic Republic of Congo and the Central African Republic. The promising results of the Phase III study of FEX were declared by DND*i* in 2017. A summary of the clinical trial data of FEX is mentioned in Table 3. The European Medicines Agency, Democratic Republic of Congo, and the United States Food and Drug Administration approved FEX for the treatment of HAT in 2018, 2019, and 2021, respectively. The drug approval process in other endemic countries is also underway. The development timeline of FEX is provided in Scheme 1 [14,17,18,19].

## 3. Pharmacology of FEX

**Scheme 2:**
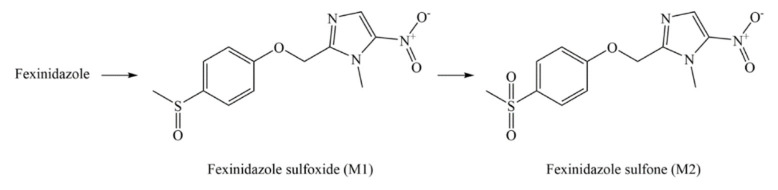
Transformation of fexinidazole into its metabolites.

The nitroheterocyclics require bio-activation of their nitro groups to become biologically active. The nitro-reductase (NTR) enzyme is shown to activate the nitroimidazoles FEX and nifurtimox in the parasites [23]. The NTR enzyme of the parasite activates FEX and its active metabolites (FEX sulfoxide and FEX sulfone) (Scheme 2) to generate reactive amines that damage the DNA and proteins of the parasite. However, the precise mechanism by which FEX, FEX sulfoxide, and FEX sulfone demonstrate action against *T. brucei* is still unknown [16]. One study has stated that the action of the NTR enzyme on nitroaromatic compounds (Nifurtimox and Benznidazole) leads to the generation of different toxic fragments (open chain nitrile and hydroxylamine) that exert their effects against *T. brucei* [24]. The chemical structure of Benznidazole is similar to FEX [24]. Therefore, the authors believe that there is a possibility that the NTR enzyme converts the nitro group of FEX to nitroso intermediate followed by the generation of hydroxylamine intermediate. The hydroxylamine intermediate may be responsible for the activity of FEX against *T. brucei*.

FEX is well absorbed orally. After oral administration, FEX rapidly converts to its active metabolites, FEX sulfoxide (M1) and FEX sulfone (M2) (Scheme 2) [16,25]. FEX tablet must be swallowed (not chewed) during or just after a solid meal (not liquid meal) to increase its absorption (2–3 times), especially for stage-2 HAT. A dose administered without food is considered as a dose skipped [16]. The apparent volume of distribution of FEX is 3222 ± 1199 L [16], whereas the protein binding of FEX, M1, and M2 are 98%, 41%, and 57%, respectively [16,25]. FEX is rapidly metabolized via hepatocytes to the M1 and M2 metabolites (Scheme 2) which provide most if not all the trypanocidal activity against *Trypanosomia brucei gambiense* strains of the parasite. The sulfone metabolite (M2) does not seem to go through further metabolism [16,22,25,26]. The elimination of FEX is almost completely extra-renal, wherein the majority of the excreted metabolites are M1 and M2 [16,25]. The reported half-life (hours) of FEX, M1, and M2 are 15.0, 16.0, and 23.0 h, respectively [16,25], whereas the clearance (liter/hour) of FEX is 161.0 [16]. The adverse effects of FEX include QT prolongation, CNS and psychiatric-related adverse reactions, risk of infection, hepatotoxicity, and disulfiram-like reactions [16]. The toxic (overdose) effects of FEX in adults (3600 mg/day for 14 days) comprise elevated levels of transaminases, panic attack, and vomiting. The safety and efficacy of FEX for the treatment of both stages of HAT have been established in children ≥ 6 years old and weight ≥ 20 kg (National clinical trial numbers 02169557 and 02184689, Table 3). However, such pediatric patients may be more sensitive to vomiting. The safety and efficacy of FEX have not been established in children <6 years old and weight <20 kg [16]. The overdose in pediatric HAT patients causes elevated potassium, reduced calcium levels, and vomiting. The treatment of FEX toxicity is supportive therapy with monitoring due to the lack of its antidote [16,22]. The metabolism of FEX involves many enzymes including CYP1A2, CYP2B6, CYP2C19, CYP2D6, CYP3A4, CYP3A5, and flavin mono-oxygenase-3 (FMO-3). Therefore, drugs, including herbal medicines, which are metabolized by these enzymes may interfere with the FEX treatment [16].

In clinical trials, a difference in the efficacy of FEX has been detected in both stages of HAT (National clinical trial number 01685827; Table 3). In the first stage of HAT, a treatment success rate of 98.7% and 97.6% was observed. However, a decrease in the efficacy of FEX (86.9%) was seen in a comparison of nifurtimox-eflornithine combination therapy (98.7%) in the second stage of HAT. Furthermore, all-cause mortality was higher in FEX treated patients (4.4%) than nifurtimox-eflornithine combination therapy (0%). Accordingly, the patients of the second stage of HAT can be treated with FEX in the absence of other treatment options like nifurtimox-eflornithine combination therapy [16]. The safety and efficacy of FEX have not been fully established in certain populations (children below 6 years, pregnant/lactating women, and hepato-compromised patients) [16].

## 4. Clinical Trials on FEX

A search on the clinicaltrial.gov database [27] was done utilizing keywords “fexinidazole, HOE-239, and HOE239”. A total of 12 clinical studies were found, which are listed in Table 3.

**Table 3:** Interventional clinical trials on fexinidazole.

Condition	Phase (Number Enrolled)	Status (Study Start Date (SSD); Study Completion Date (SCD); Last Update Date (LUD))	National Clinical Trial (NCT) Number/Other IDs (Sponsor/Collaborators; Funder Type; Location)
Chagas Disease and South American Trypanosomiasis	Phase 2 (140)	Unknown (SSD: July 2014; SCD: February 2016; LUD: 15 July 2015)	NCT02498782/DNDi-CH-FEXI-001 (Drugs for Neglected Diseases initiative; Other; Bolivia)
Trypanosomiasis (African)	Phase 1 (30)	Terminated (SSD: September 2011; SCD: February 2012; LUD: 31 March 2017)	NCT01483170/DNDiFEX003 (Drugs for Neglected Diseases initiative; Other; France)
Visceral Leishmaniasis	Phase 2 (14)	Terminated (SSD: November 2013; SCD: September 2015; LUD: 30 October 2015)	NCT01980199/FEXI VL001 (Drugs for Neglected Diseases initiative; Other; Sudan)
r-Human African Trypanosomiasis	Phase 2/3 (50)	Recruiting (SSD: 29 September 2019; SCD: March 2023; LUD: 30 August 2021)	NCT03974178/DNDi-FEX-07-HAT (Drugs for Neglected Diseases initiative; Other; Malawi and Uganda)
Trypanosomiasis (African)	Phase 1 (30)	Completed (SSD: March 2015; SCD: June 2015; LUD: 8 October 2015)	NCT02571062/DNDiHATFEX008 (Drugs for Neglected Diseases initiative; Other; France)
Human African Trypanosomiasis	Phase 2/3 (394)	Completed (SSD: October 2012; SCD: 26 April 2017; LUD: 20 February 2018)	NCT01685827/DNDiFEX004 (Drugs for Neglected Diseases initiative; Other; Batangafo, Bagata, Congo, etc.)
Human African Trypanosomiasis	Phase 2/3 (125)	Completed (SSD: May 3, 2014; SCD: 27 June 2017; LUD: 24 June 2020)	NCT02184689/DNDiHATFEX006 (Drugs for Neglected Diseases initiative; Other; Congo)
Human African Trypanosomiasis	Phase 2/3 (230)	Completed (SSD: 30 April 2014; SCD: 25 April 2017; LUD: 24 June 2020)	NCT02169557/DNDiHATFEX005 (Drugs for Neglected Diseases initiative; Other; Congo)
Human African Trypanosomiasis	Phase 1 (108)	Completed (SSD: September 2009; SCD: October 2010; LUD: 6 April 2017)	NCT00982904/DNDiFEX001 (Drugs for Neglected Diseases initiative and Sanofi; Other/Industry; France)
Human African Trypanosomiasis and Trypanosomiasis (Gambian)	Phase 3 (174)	Completed (SSD: 17 November 2016; SCD: 1 February 2021; LUD: 11 October 2021)	NCT03025789/DNDi-FEX-09-HAT (Drugs for Neglected Diseases initiative and Sanofi; Other/Industry; Congo, Mbuji-Mayi, Bagata, etc.)
Pharmacokinetic in Healthy Volunteers	Phase 1 (12)	Completed (SSD: February 2011; SCD: April 2011; LUD: 31 March 2017)	NCT01340157/DNDiFEX002 (Drugs for Neglected Diseases initiative and Sanofi; Other/Industry; France)
Chagas’ Disease (Chronic)	Phase 2 (45)	Completed (SSD: 13 November 2017; SCD: 28 August 2019; LUD: 23 September 2020)	NCT03587766/DNDi-FEX-12-CH (Drugs for Neglected Diseases initiative; Other; Spain)

It is evident from Table 3 data that the Drugs for Neglected Diseases initiative is developing FEX for the treatment of other neglected diseases also (Chagas disease, South American Trypanosomiasis, Leishmaniasis, and Trypanosomiasis). These clinical trials indicate the foreseeable potential of FEX to treat many neglected diseases, and make FEX an important molecule for neglected diseases.

## 5. Patent Searching

**Scheme 3:**
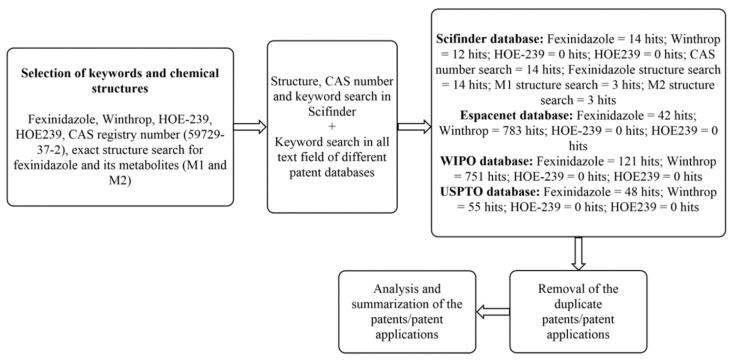
Patent searching methodology.

The patents searching was done on 6 November 2021, utilizing different patent databases, namely, Espacenet [28], USPTO [29], Patentscope [30], and Sci-finder [31]. The patent searching methodology is described in Scheme 3. The bibliographic data of the patents/patent applications (Table 4) was obtained from Espacenet, Patentscope, and USPTO. The patents/patent applications that explicitly or implicitly cover FEX or any of its synonyms in the claim section were included in this review and analyzed.

## 6. Patent Analysis

### 6.1. Compound Patent

**Figure 2:**
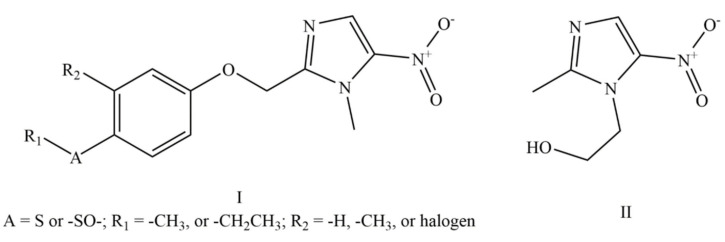
Compounds claimed in US4042705A (**I**) and structure of metronidazole (**II**).

US4042705A claims 1-methyl-2-(phenyloxymethyl)-5-nitro-imidazole derivatives of formula I (Figure 2), which generically covers FEX and FEX sulfoxide (M1 metabolite of FEX) [21]. These compounds were developed based on the chemical structure of metronidazole of formula II (Figure 2). US4042705A does not provide any experimental data about the antiprotozoal activity of any compound. However, it claims a pharmaceutical composition (oral or local route) of the claimed compound (Figure 2) for treating protozoal diseases as well as diseases caused by bacteria and fungi in admixture or conjunction with a pharmaceutically acceptable carrier and/or excipient.

**Scheme 4:**
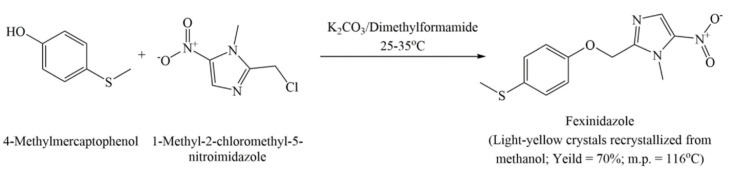
Method of preparing fexinidazole provided in Example 1 (Method a) of US4042705A.

**Scheme 5:**
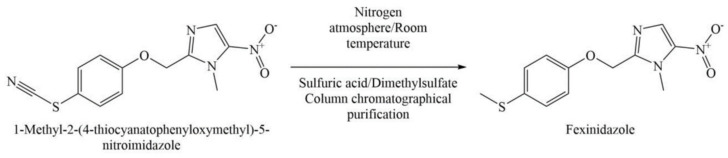
Method of preparing fexinidazole provided in Example 3 (Method b) of US4042705A.

This patent provides two methods for preparing FEX, which have been summarized in Scheme 4 and Scheme 5. As per Scheme 4, FEX is synthesized by mixing a solution of 4-methylmercapto-phenol (0.1 mol; 14 g) in dimethylformamide (DMF) with potassium carbonate powder (0.1 mol, 13.8 g). To this mixture is added, a solution of 1-methyl-2-chloromethyl-5-nitro-imidazole (0.1 moL, 17.6 g) in DMF gradually with stirring at an ambient temperature. The resulting mixture is stirred for 1 hr, maintaining the temperature of this exothermic reaction below 35 °C. The precipitate obtained after pouring the reaction mixture over crushed ice is filtered and crystallized with methanol/water to obtain a light yellow crystalline solid in 70% yield. FEX can alternatively be prepared from 1-methyl-2-(4-thiocyanatophenyloxymethyl)-5-nitro-imidazole. The starting material is added slowly to the mixture of concentrated sulfuric acid and dimethyl sulfate under nitrogen atmosphere at room temperature and then kept aside for 12 h followed by heating at 60 °C for 30 min. The resulting mixture is poured over crushed ice to yield precipitates which can be purified using column chromatography to obtain pure crystals of FEX.

**Scheme 6:**
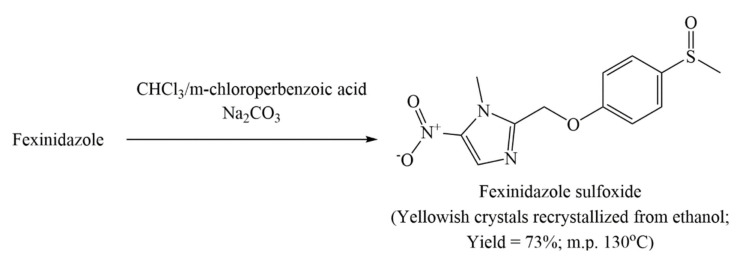
Method of preparing fexinidazole sulfoxide (M1 metabolite of fexinidazole) provided in Example 2 (oxidation) of US4042705A.

US4042705A also mentions one method for preparing FEX sulfoxide which has been depicted in Scheme 6 [21]. A solution of FEX (0.1 mol; 27.9 g) in chloroform (200 mL) is added slowly to a solution of m-chloroperbenzoic acid (0.1 moL; 17.3 g) in chloroform and then stirred for 60 min at room temperature. Dilute sodium carbonate solution is added to the reaction mixture and the organic layer is separated, dried over anhydrous sodium sulfate, and finally evaporated to obtain a residue which upon recrystallization with ethanol produces pure yellow crystals of 1-methyl-2-(4-methyl-sulfinylphenyl-oxymethyl)-5-nitro-imidazole in 73% yield.

### 6.2. Process Patents

CA1079738A claims the preparation of FEX sulfone metabolite (M2) by the oxidation of FEX employing an oxidizing agent (hydrogen peroxide, peracetic acid, pertrifluoroacetic acid, chloroperbenzoic acid, nitric acid, chromic acid, chromic acid anhydride, permanganates, hypochlorites, chlorates, perchlorates, periodates, and nitric oxides) and an inert solvent (acetic acid, trifluoroacetic acid, methylene chloride, or chloroform) or dispersing agent at a temperature of 0–100 °C [32]. Example 1 of CA1079738A provides preparation of FEX sulfone metabolite (M2) (yellowish crystals recrystallized from isopropanol; Yield = 93%; m.p. = 157 °C) from FEX using 35% hydrogen peroxide solution and acetic acid at a temperature ranging from room temperature to 60 °C. The patent states that M1 can also be converted to M2 utilizing the same reaction, but employing half the amount of the oxidizing agent [32].

**Scheme 7:**
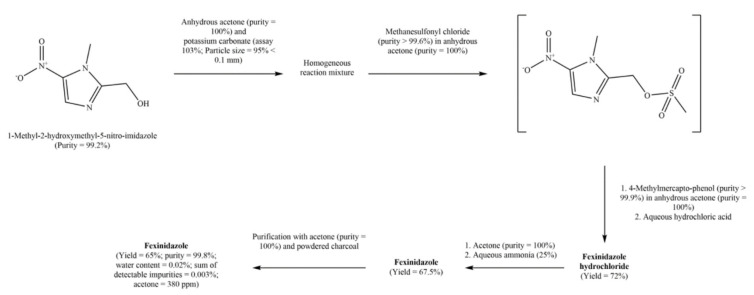
Method of preparing fexinidazole provided in Example 2 (oxidation) of US9758488B2.

**Figure 3:**
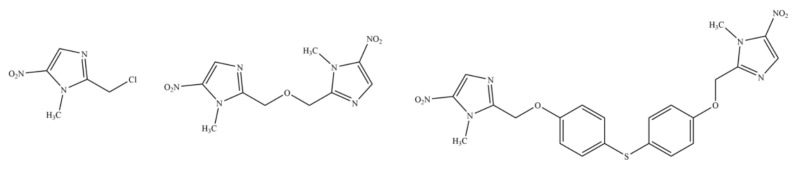
Structures of the process-related impurities mentioned in US9758488B2.

US9758488B2 discloses a method of preparing FEX (Scheme 7) with a limited number and amounts of process-related impurities (Figure 3). The patent also discloses hydrochloride salt of FEX [33]. According to this method, FEX of high purity grade and in substantial yield in industries can be obtained in four simple and easy steps. In step 1 of this nucleophile substitution reaction, 1-methyl-2-hydroxymethyl-5-nitro-imidazole is reacted with a solution of methanesulfonyl chloride in acetone. The reaction mixture is stirred at room temperature in the presence of a suspension of powdered alkaline carbonate in an anhydrous polar aprotic organic solvent such as acetone or acetonitrile to produce the intermediate 1-methyl-5-nitro-1H-imidazol-2-yl)methyl methanesulfonate. Step 2 involves the reaction of the resulting reaction mixture with a solution of 4-methyl-mercapto-phenol in anhydrous acetone. After stirring, heating, and quenching with water, the acetone phase is separated and mixed with aqueous hydrochloric acid over 1 h to obtain FEX hydrochloride salt (72% yield) in step 3. Finally, FEX hydrochloride salt is converted into a free FEX base by neutralization with an aqueous ammonia solution. The process of this patent is distinct from the prior art process because a single solvent (acetone) is used in all the steps; a single catalyst (potassium carbonate) is used in the first two steps; synthesis of FEX hydrochloride takes place in one step, and the FEX is obtained in good yield and purity. Two processes for preparing FEX are also provided in non-patent literature [34,35]. Since our review focuses on patent literature, we have not discussed it here in detail.

### 6.3. Patents/Applications Related to the Method of Treatment

US9585871B2 claims a method for curing a canine animal of a *Leishmania infantum* parasite infection employing an effective amount of FEX, which is metabolized in the canine animal to produce a parasiticidal effective amount of FEX sulfone (M2) and sulfoxide (M1) metabolites that are responsible for killing the *Leishmania infantum* parasite [36]. The metabolites are present in the canine animal’s plasma in concentrations exceeding 2 ng/mL for at least one hour of each day throughout the FEX administration. The invention demonstrated that FEX treatment can substantially eliminate or cure *L. infantum* infection in canines. No relapse was observed 180 days after treatment initiation. The patent exemplifies the efficacy of FEX in dogs Infected with *L. infantum* [36].

WO9912547A1 states that bio-reductive compounds are a class of compounds requiring metabolic reduction to generate cytotoxic metabolites [37]. This patent application claims the use of a bio-reductive compound (FEX, metronidazole, benznidazole, tinidazole, ornidazole, misonidazole, secnidazole, carnidazole, nimorazole, panidazole, or satranidazole) capable of targeting tissues having an enhanced reductase activity in the manufacture of a medicament for use in the treatment of rheumatoid or osteoarthritis, Crohn’s disease, or periodontitis. The bio-reductive compound can also be used in combination with a second medicament such as non-steroidal anti-inflammatory agent (NSAID), a corticosteroid or glucocorticoid, an alkylating agent, an antimalarial, a gold compound, penicillamine, sulphasalazine, methotrexate, or azathioprine [38]. WO9912548A1 [38] is a family member of WO9912547A1 [36]. It also claims the use of a bioreductive compound in the manufacture of a medicament for use in the treatment of an inflammatory condition associated with hypoxia, ischemia, diabetes, stroke, sepsis, Alzheimer’s disease, cancer, kidney disease, digestive diseases, liver disease, tissue transplantation, the healing of wounds, fibrotic disorders, cardiovascular reperfusion injury, cerebral reperfusion injury, cystic fibrosis, psoriasis, para-psoriasis, ulcers, AIDS, and inflammatory bowel disease [38].

US2021220335A1 claims a method for the prevention and/or treatment of an infectious disease (diarrhea, colitis) using a nitroimidazole (metronidazole, bamnidazole, carnidazole, dimetridazole, FEX, ipronidazole, ornidazole, panidazole, secnidazole, ternidazole, tinidazole), wherein the infectious disease is caused by bacteria from the genus Clostridium (antibiotic-resistant bacterial strain) [39]. The nitroimidazole can also be co-administered with a berberine alkaloid for a synergistic effect. The patent application provides biological activity data of metronidazole and berberine, but it is silent about the biological activity data for FEX and its combination with berberine [39].

### 6.4. Patents/Applications Related to the Combination of FEX with Other Molecules

WO2017072523A1 claims use of delamanid and its derivatives alone or in combination with one or more additional compounds of therapeutic utility (FEX, FEX sulfone, nifurtimox, etc.) to treat parasitic disease caused by or associated with parasites selected from the order Kinetoplastida consisting of species belonging to the genera Leishmania and Trypanosoma including visceral leishmaniasis, mucocutaneous leishmaniasis, cutaneous leishmaniasis, African trypanosomiasis, and Chagas’ disease [40]. This patent application does not exemplify the treatment of any parasitic diseases using a combination of delamanid and its derivatives with FEX [40].

US10392363B2 [41], US10562880B2 [42], and US10752606B2 [43] are members of the same patent family. US10392363B2 claims a method of treating or preventing protozoan colonization or infection (trypanosomosis, amoebiasis, Chagas disease, giardiasis, leishmaniasis, malaria, toxoplasmosis, etc.) using a therapeutically effective amount of carbonimidic dihydrazides and their salt alone or in combination with an antiprotozoal agent like FEX [41]. US10562880B2 claims a method of treating or preventing a protozoan infection using a therapeutically effective amount of a pyrimidine derivative alone or in combination with nitroimidazoles (FEX) [42]. US10752606B2 claims a method of treating a protozoan infection using NCL258 and NCL261 alone or in combination with nitroimidazoles (FEX) [43]. The US10392363B2 [41], US10562880B2 [42], and US10752606B2 [43] do not provide any experimental data about the efficacy of the combination of carbonimidic dihydrazides, pyrimidine derivatives, NCL258, and NCL261 in combination with FEX.

WO2019244049A1 covers cyanotriazoles compounds to treat kinetoplastid diseases (HAT, Chagas disease, and leishmaniasis) alone or in combination with meglumine antimoniate, stibogluconate, amphotericin, miltefosine, paromomycin, pentamidine, suramin, melarsoprol, eflornithine, FEX, acoziborole, benznidazole, nifurtimox, and amphotericin-B. This patent application does not provide any experimental evidence for the biological effects of the combination of cyanotriazoles compounds and FEX [44].

### 6.5. Patents/Applications Related to Novel Compositions of FEX

WO2019043701A1 claims a composition for treating a parasitic infectious disease or disorder, wherein the composition contains a metal oxide (iron oxide, aluminum oxide, zinc oxide, copper oxide, manganese oxide,), and a shell comprising an ion of a lanthanide element (cerium, lanthanum, praseodymium, neodymium, promethium, samarium, europium, gadolinium, terbium, dysprosium, holmium, erbium, thulium, ytterbium, lutetium) linked to a polymer (polyethylenimine (PEI), poly-L-lysine, chitosan, poly-L-arginine, dendrimeric polyamidoamine (PAMAM), polyacrylate, polyphosphate, polyethyleneglycol (PEG)) and an active agent (pentamidine, suramin, melarsoprol, eflornithine, nifurtimox, benznidazole, FEX, oxaborole, pafuramidine, doxorubicin, amphotericin B). This patent application does not exemplify any biological activity data of the composition comprising FEX [45].

US2021322329A1 claims a lipid nanoparticle-based pharmaceutical composition containing particles of one or more active substances (benzimidazole, nifurtimox, ertanidazole, buthionine sulfoximine, eflornithine, melarsoprol, pentamidine, suramin, and FEX) for the treatment of diseases produced by trypanosomes and for the treatment of tumors of neural origin. This patent application is silent about the biological effect of the lipid nanoparticles of FEX [46].

US2021052498A1 claims a nanocarrier for treating Chagas disease comprising a poly(ethylene glycol)-block-poly(propylene sulfide) copolymer; and a therapeutic agent (benznidazole, nifurtimox, FEX) for treating Chagas disease and *Trypanosoma cruzi* infection [47]. The patent application states that the subject receiving such nanocarrier formulation does not experience one or more side effects associated with the therapeutic agent because the therapeutic agent can be administered at substantially lower levels than the free form drug. The patent application exemplifies the biological effects of the nanocarrier of benznidazole, but is silent about the effects of the nanocarrier of FEX [47].

WO2021123775A2 relates to engineered platelets with chimeric platelet receptors (CPR) with the desired target specificity. Furthermore, the engineered platelets may encompass a therapeutic agent which may be released upon activation of the platelet. The patent application mentions the names of hundreds of therapeutic agents including FEX. However, this application does not provide any experimental data for FEX [48].

US9016221B2 claims a polymeric article having a surface topography for resisting bioadhesions of organisms comprising a biologically active agent [49]. The claims mention a large number of biologically active agents (antibiotics, anti-proliferative/anti-mitotic/alkylating agents, antiplatelet agents, anti-metabolites, anti-coagulants, anti-migratory agents, antisecretory agents, anti-inflammatory agents, and anti-sense oligonucleotides) including FEX. However, no example of a polymeric article comprising FEX has been provided in the patent. The claimed polymeric article may find utility in biomedical implants, biomedical instruments, hospital surfaces, clothing/protective personal wear, biomedical packaging, cleanroom surfaces, food packaging, food preparation surfaces, etc. [49].

## 7. Conclusions

The approval of the orally effective FEX tablets by EMA and USFDA is a landmark for the treatment of HAT. Some patents/patent applications related to salt, process, method of treatment, drug combinations, and compositions of FEX have been filed. However, there remains a great scope to develop more inventions based on FEX for the treatment of other protozoal and microbial infections. New combinations of FEX with other treatments of HAT may also provide fruitful results. The development of FEX has motivated scientists to develop more oral treatments and vaccines against HAT and other protozoan diseases. Accordingly, new oral treatments for HAT are also anticipated in the future.

## 8. Expert Opinion

HAT is caused by two subspecies of *T. brucei* called *T.b. gambiense* (causes gHAT) and *T.b. rhodesiense* (causes rHAT). This disease is prevalent in sub-Saharan countries. The gHAT is a chronic illness of endemic areas and constitutes about 85–90% of the total cases of HAT. The rHAT is an acute systemic illness of local populations/travelers and constitutes about 10–15% of the total cases of HAT. Only a few patient non-compliant treatments (mainly IV treatments) were available for this illness until 2018 [50]. None of the treatments was effective against both stages (stage-1 and stage-2) of HAT [50,51]. The efforts to develop oral and patient-compliant interventional therapy have resulted in the development of FEX. The EMA approved FEX in 2018 as the first all-oral treatment for HAT, which could be used in both stages of HAT. FEX became part of WHO’s list of essential drugs in 2019, and USFDA also approved FEX in 2021 (Figure 1).

**Figure 4:**
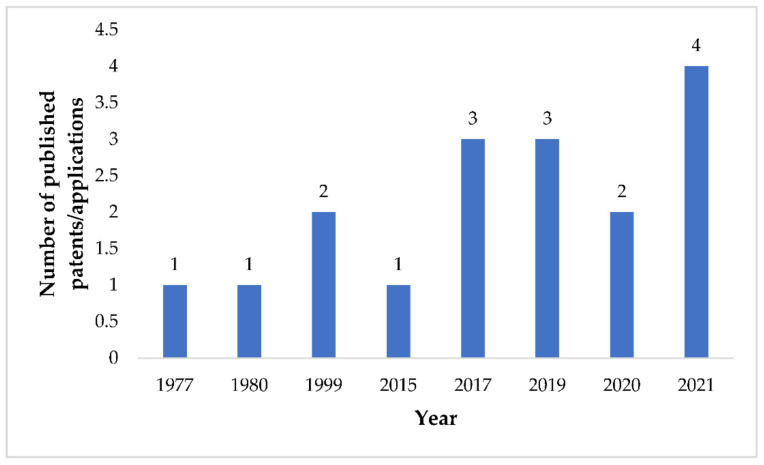
Publication years of the patents/patent applications of fexinidazole.

The patent searching of FEX resulted in the identification of 17 patents/patent applications belonging to 14 patent families (Table 4). It can be observed that no patent/patent application related to FEX was published from 1980–1998 and 2000–2014 (Figure 4). This may be because, during 1980–1998, the development of FEX was not part of the strategic planning of Sanofi/Hoechst AG, and during 2000–2014 FEX was in the initial phase of the clinical trial for an orphan disease.

**Figure 5:**
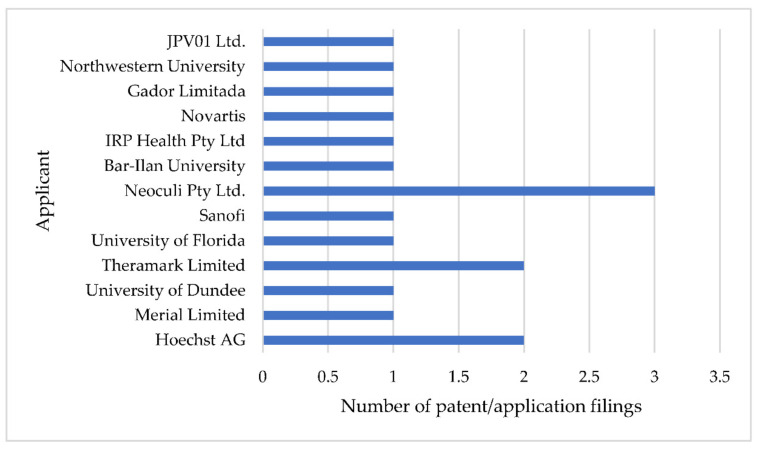
Number of patent application filings by different applicants.

It is quite interesting to note that Sanofi/Hoechst, the innovator of FEX, has only three patents to its credit (Table 4) (Figure 5). The first patent was the compound patent of FEX, which has expired [21]. The second expired patent belongs to a process for preparing FEX-sulfone (M2) [32]. The third patent relates to a process for preparing FEX, which is still enforceable [33]. The majority of the published patents/patent applications were filed in Europe (Germany and United Kingdom) followed by the USA and Australia (Table 4).

**Figure 6:**
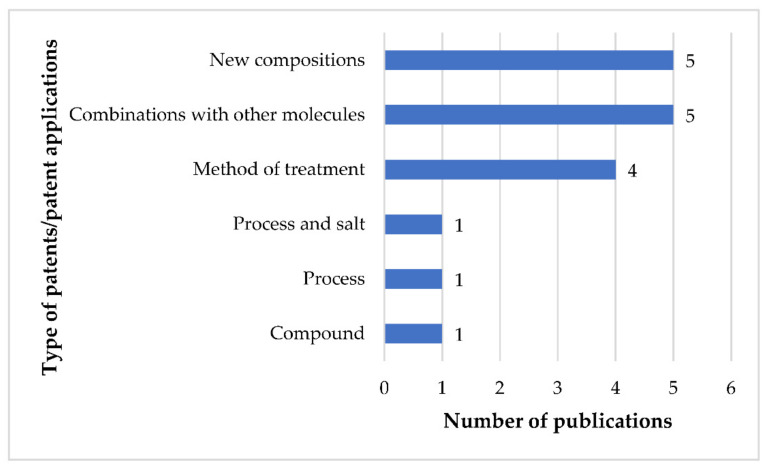
Types patents/patent applications of fexinidazole and the numbers of their corresponding publications.

The identified 17 patents/patent applications of 14 patent families claimed different inventions of FEX (Figure 6). The compound patent of FEX [21] has expired long ago and is also not listed in the orange book of the USFDA [52,53]. Since this patent has expired, it is not eligible for any patent term extension [54,55].

Accordingly, we trust that the generic entry in the USA will be after the expiry of the orphan drug exclusivity (16 July 2028) (Table 2). Our search revealed one enforceable patent related to the process for preparing FEX [33]. However, it can easily be designed around by following the process for preparing FEX provided in the compound patent [21]. One patent discloses the hydrochloride salt of FEX [33]. This indicates that acid addition salts of FEX are possible, and can be developed to check their utility as a therapeutic agent and to develop a novel dosage form of FEX. The base of FEX shows polymorphism, wherein crystalline Form-I is known [20]. Accordingly, patent filings related to new salts and polymorphs of FEX are foreseeable. Some patents/patent applications claiming the use of FEX to treat kinetoplastid diseases, inflammatory diseases, and other microbial infections alone or in combination with other compounds were also identified [36,37,38,39,40,41,42,43,44]. However, most of these patents/patent applications did not provide any experimental data to support their claims. Accordingly, we foresee a scope to carry out such experiments to develop a new combination of FEX with other therapeutic agents. It has been observed that most of the treatments are given by IV routes. There is a possibility to make new oral dosage forms of such treatments with fewer side effects, for example, the drugs may be encapsulated into a cyclodextrin molecule. This strategy has been used on melarsoprol [56]. The cyclodextrin complex of FEX in combination with other IV HAT treatments may provide promising results. FEX is not considered as the first-line treatment of the second stage of HAT in the presence of nifurtimox-eflornithine combination therapy [16]. Accordingly, the authors see a patentable opportunity to identify a novel drug combination therapy of FEX that may be a patient compliant therapy for the patients suffering from the second stage of HAT. The authors also foresee the development of FEX to treat Chagas disease and Leishmaniasis along with other neglected diseases (Table 3).

Agencies like DNDi are committed to developing drugs for neglected diseases. This agency has many drugs for HAT (SCYX-13330682, SCYX-1608210, and Acoziborole), Leishmaniasis (DNDI-6174, DNDI-6148, DNDI-0690, GSK899, DDD853651, LXE408, CpG-D35, GSK245, and DDD1305143), Chagas disease (New benznidazole regimens), Filaria (CC6166, Oxfendazole, Emodepside, and TylAMac), and Mycetoma (Fosravuconazole) in the pipeline [57,58]. All these pipelines for the neglected diseases have been possible because of the strategies (drug repurposing, collaborative drug discovery, and multidisciplinary interventions) employed to identify the lead compounds [59]. The DNDi in collaboration with Sanofi has also delivered orally active FEX for clinical use to treat HAT [60].

HAT has been targeted by the WHO, which is committed to terminating its transmission by 2030. The efforts of the WHO have led to a promising decrease in the number of cases at the global level [2,58,61]. Some gaps still exist and need to be addressed to fulfill the commitment of the WHO. These gaps include eliminating the transmission of tsetse flies through asymptomatic, but infected domestic animals and travelers [61]. The authors trust that the introduction of FEX therapy is a welcome advancement for HAT treatment, which in combination with the vector control would help to eliminate HAT in the future.

## Figures and Tables

**Table 1:** Current treatment option for human African trypanosomiasis.

Drug (Dosage Form and Administration)	Comments
Treatment of Stage 1 of Human African Trypanosomiasis	
Pentamidine (Solution for inhalation/Injection)	A skilled and trained professional is needed for drug administration. It is administered as a single daily intramuscular/intravenous injection for seven days. It can cause severe hypotension after intramuscular/intravenous administration, hypoglycemia, acute pancreatitis, and cardiac arrhythmias, and is effective against stage-1 of g-human African trypanosomiasis only because it does not cross the blood–brain barrier efficiently [9].
Suramin (Intravenous, injection)	A skilled and trained professional is needed for drug administration. It is mainly used for stage-1 of r-human African trypanosomiasis, and rarely used for stage-1 g-human African trypanosomiasis. It can cause renal toxicity and anaphylactic reactions [10].
Treatment of Stage 2 of Human African Trypanosomiasis	
Nifurtimox (Tablet, Oral)	The combination of nifurtimox with eflornithine is the first-line treatment for stage-2 of human African trypanosomiasis. It has potential for genotoxicity, carcinogenicity, fetal toxicity, worsening of neurological and psychiatric conditions, hypersensitivity, decreased appetite and weight loss, and porphyria [11].
Eflornithine (Intravenous, injection)	A skilled and trained professional is needed for drug administration and requires long therapy. It can cause fever, pruritus, hypertension, cough, anorexia, nausea, vomiting, diarrhea, abdominal pain, headaches, and is the second-line treatment for stage-2 of g-human African trypanosomiasis [12].
Melarosoprol (Intravenous, injection)	A skilled and trained professional is needed for drug administration and is effective for stage-2 g-human African trypanosomiasis. Its administration is painful and toxic. The adverse events may be life-threatening including encephalopathic syndrome [13].
Nifurtimox-eflornithine combination therapy (Oral Nifurtimox + Intravenous Eflornithine)	A skilled and trained professional is needed for drug administration. It needs systematic hospitalization and is mainly used for stage-2 of g-human African trypanosomiasis [14].

**Table 2:** Product details of the USFDA approved Fexinidazole.

Proprietary Name (Application Number; Applicant)	Dosage Form; Route; Strength	Marketing Status (Exclusivities)	Recommended Dosage	
			Greater than or Equal to 35 kg	Greater than or Equal to 20 and Less than 35 kg
Fexinidazole (N214429; Sanofi Aventis)	Immediate release tablet; Oral; 600 mg	Prescription (New Chemical Entity (NCE) and Orphan Drug Exclusivity (ODE-359) expiring on 16 July 2026, and 16 July 2028, respectively)	Loading dose (1800 mg, 3 tablets) for four days followed by the maintenance dose (1200 mg, 2 tablets) for 6 days	Loading dose (1200 mg, 2 tablets) for four days followed by the maintenance dose (600 mg, 1 tablet) for 6 days

**Table 4:** Bibliographic data of the cited patents/patent applications.

Patent/Patent Application Number (Assignee; Publication Date; Priority Country; Estimated Expiry Date)	Legal Status on 6 November 2021 (International Patent Classification)	Family Members on 6 November 2021
US4042705A (Hoechst AG; August 16, 1977; Germany; Expired)	Expired patent (A61K31/415, A61P31/04, C07D233/94, (IPC1-7): A61K31/415, C07D233/94)	AT359059B, AU500640B2, CH605813A5, CH605819A5, DK142412C, EG12284A, ES449589A1, FI61185C, FR2317925B1, GB1541280A, GR66090B, HU173463B, IE43692B1, IL50023A, IT1064924B, JPS5231074A, LU75359A1, MX3539E, MY8100120A, NL7607495A, NO145136B, NO145136C, NZ181421A, PT65350B, SE414927B
CA1079738A (Hoechst AG; 17 June 1980; Germany; Expired)	Expired patent (C07D233/94, (IPC1-7): C07D233/94)	AT361468B, ATA128477A, CH624942A5, DK144524C, EG13828A, ES456117A2, FI770599A, IT1115608B, LU76834A1, NL7701838A, NO770648L, SE7702126L
US9758488B2 (Sanofi; 12 September 2017; Europe; 24 December 2032)	Patented case (C07D233/94)	AP3759A, BR112015011446A2, CA2892334C, CN104797562B, CR20150286A, CY1118983T1, ECSP15025960A, EP2922822B1, ES2618800T3, HRP20170390T1, HUE032160T2, IL238934A, MX365587B, PL2922822T3, SG11201504047TA, SI2922822T1, WO2014079497A1, ZA201503063B
US9585871B2 (Merial Limited; 7 March 2017; USA; 31 January 2034)	Patented case (A61K31/4164)	AU2014212217B2, CY1120381T1, DK2950795T3, EP2950795B1, ES2681420T3, HK1211466A1, HRP20181181T1, HUE039467T2, IL240246A, LT2950795T, MX367952B, PL2950795T3, PT2950795T, RS57469B1, SI2950795T1, WO2014121064A1
WO9912547A1 (Theramark Limited; 18 March 1999; United Kingdom; 8 September 2018)	Lapsed (A61K31/404, A61K31/407, A61K31/4155, A61K31/4174, A61K31/535, (IPC1-7): A61K31/40, A61K31/41, A61K31/415, A61K31/535)	AU9082698A, AU9082798A
WO9912548A1 (Theramark Limited; 18 March 1999; United Kingdom; 8 September 2018)		
US2021220335A1 (IRP Health Pty Ltd.; 22 July 2021; Australia; 29 May 2039)	Under examination (A23K20/121, A23K20/137, A23K20/195, A23K50/30, A23L33/00, A23L33/10, A61K31/4164, A61K31/4375, A61K9/00, A61P31/04)	AU2019277198A1, CN112543635A, EP3810137A1, WO2019227149A1
WO2017072523A1 (University of Dundee; 4 May 2017; United Kingdom; 28 October 2036)	Lapsed (A61K31/454, A61P33/02)	None
US10392363B2 (Neoculi Pty Ltd.; 27 August 2019; Australia; 28 August 2035)	Patented case (A61K31/155, A61K31/381, A61K31/404, A61K31/496, A61K31/505, A61K45/06, A61K9/00, C07C281/18, C07C335/40, C07D209/08, C07D239/50, C07D295/135, C07D333/58, A61K31/17, A61K31/341, A61K31/4045, A61K31/4192, A61K31/44, A61K31/498, C07C211/29, C07C251/24, C07C47/565, C07D209/14, C07D239/48, C07D241/20, C07D249/14, C07D251/54, C07D307/56, C07D311/58, C07D401/14)	AU2015311598A1, AU2020204441B2, BR112017004153A2, CA2959440A1, CN107106524B, EP3188722B1, JP2017528518A, RU2719593C2, RU2020113652A, US10829468B2, US2021009552A1, WO2016033635A1
US10562880B2 (Neoculi Pty Ltd.; 18 February 2020; Australia; 28 August 2035)		
US10752606B2 (Neoculi Pty Ltd.; 25 August 2020; Australia; 28 August 2035)		
WO2019244049A1 (Novartis; 26 December 2019; USA; 18 June 2039)	National phase entry in many countries (A61K31/4196, A61K31/4439, A61P33/02, C07D401/14, C07D403/06)	AU2019291490A1, BR112020025538A2, CA3100954A1, CL2020003252A1, CN112313217A, CR20200619A, CU20200102A7, EA202190064A1, ECSP20080991A, EP3810598A1, JOP20200327A1, JP2021528397A, KR20210022646A, MA52977A, PE20210780A1, PH12020552186A1, SG11202012628XA
WO2019043701A1 (Bar-Ilan University; 7 March 2019; USA; 28 August 2038)	Entered into Europe (A61K31/155, A61K31/7105, A61K33/26, A61K38/00, A61K9/14, B82Y5/00, C01G49/02, C12N15/113)	EP3675875A1
US2021322329A1 (Gador Limitada; 21 October 2021; Chile; 21 August 2038)	Under examination (A61K31/541, A61K9/51, A61P33/02)	CA3113862A1, EP3842029A1, WO2020037438A1
US2021052498A1 (Northwestern University; 25 February 2021; USA; 29 July 2040)	Under examination (A61K31/4168, A61K47/34, A61K9/107, A61K9/127, A61P33/02)	None
WO2021123775A2 (JPV01 Ltd.; 24 June 2021; United Kingdom; 16 December 2040)	No national phase entry (C07K14/705, C07K16/28)	None
US9016221B2 (University of Florida Research Foundation; 28 April 2015; USA; 24 November 2025)	Patented case (A41D31/00, A61F2/02, A61L2/02, A61L27/00, B08B17/02, B08B17/06, B63B59/04, B64D15/00, A61F2/00, A61F2/12, A61F2/24, B63B1/36	None

## Data Availability

This study did not report any data.
